# Bactericidal efficacy of plasma-activated water against *Vibrio parahaemolyticus* on *Litopenaeus vannamei*

**DOI:** 10.3389/fnut.2024.1365282

**Published:** 2024-03-07

**Authors:** Huanlan Zhang, Jie Wei, Hongjie Xv, Imran Khan, Qinxiu Sun, Xihong Zhao, Jialong Gao, Shucheng Liu, Shuai Wei

**Affiliations:** ^1^College of Food Science and Technology, Guangdong Ocean University, Guangdong Provincial Key Laboratory of Aquatic Product Processing and Safety, Guangdong Province Engineering Laboratory for Marine Biological Products, Guangdong Provincial Engineering Technology Research Center of Seafood, Key Laboratory of Advanced Processing of Aquatic Product of Guangdong Higher Education Institution, Zhanjiang, China; ^2^Department of Food Science and Technology, The University of Haripur, Haripur, Pakistan; ^3^School of Environmental Ecology and Biological Engineering, Wuhan Institute of Technology, Wuhan, China; ^4^Collaborative Innovation Center of Seafood Deep Processing, Dalian Polytechnic University, Dalian, China

**Keywords:** plasma-activated water, *Vibrio parahaemolyticus*, inactivation, *Litopenaeus vannamei*, cell membrane

## Abstract

In this study, the antimicrobial mechanism of plasma-activated water (PAW) against *Vibrio parahaemolyticus* and the effectiveness of PAW in artificially contaminated *Litopenaeus vannamei* were investigated. The results demonstrated a significant reduction (*p* < 0.05) in viable counts of *V. parahaemolyticus* with increasing plasma discharge time (5, 10, 20, and 30 min) and PAW immersion time (3, 5, 10, 20, and 30 s). Specifically, the count of *V. parahaemolyticus* decreased by 2.1, 2.7, 3.3, and 4.4 log CFU/mL after exposed to PAW 5, PAW 10, PAW 20, and PAW 30 for 30 s, respectively. Significant cell surface wrinkling, accompanied by notable nucleic acid and protein leakage were observed after treatment with PAW. The permeability of the inner and outer cell membranes was significantly increased (*p* < 0.05), along with an increase in electrical conductivity (*p* < 0.05). The reactive oxygen species (ROS) within *V. parahaemolyticus* cells were significantly increased (*p* < 0.05), while superoxide dismutase (SOD) activity, and the relative expression of the *ompW*, *emrD*, and *luxS* genes were significantly decreased (*p* < 0.05). A reduction number of 1.3, 1.8, 2.1, and 2.2 log CFU/g of *V. parahaemolyticus* in artificially contaminated *L. vannamei* was obtained with PAW for 5 min. The study elucidated that PAW could destroy cell membranes, leading to cell death. The findings would strengthen strategies for *V. parahaemolyticus* control and provide a potential application of PAW for preserving aquatic products.

## 1 Introduction

Foodborne pathogens have consistently been a significant concern for the safety of fresh aquatic products (1). *Vibrio parahaemolyticus*, a Gram-negative halophilic bacterium, is commonly found in marine and freshwater products, such as shrimp, crabs, and oysters, which causes diseases in mariculture, leading to significant economic losses in the aquaculture industry (2). Furthermore, it is also the leading cause of seafood-related infections and deaths globally, posing a significant threat to food safety and human health (3). In optimal conditions, *V. parahaemolyticus* is a rapid-growing pathogen that could form biofilms, which is difficult to remove from the food chain. Therefore, it is crucial to find a safe and effective method to prevent or eliminate *V. parahaemolyticus* contamination during food processing and transportation.

In recent years, non-thermal sterilization technologies have attracted more attentions due to their low operating temperature and maintaining the quality of food (4, 5). For instance, ultraviolet, ultrahigh pressure, electrolyzed water (6, 7), pulsed electric field (8), and cold plasma (9), have been widely applied for controlling foodborne pathogenic bacteria in aquatic products. However, these methods are unable to overcome the irregularities in the food material, and the effective sterilizing substances are unable to penetrate the tiny hollows and slots in the food material, thus reducing the effectiveness of sterilization (10). Therefore, an alternative method is required to overcome these problems.

Plasma-activated water (PAW) is considered as a promising alternative non-thermal application of plasma technology, which is produced by treating water using plasma-generation devices (11). It is capable of sterilizing through oxidative stress and physical effects by interacting with active substances in PAW (12). Unlike other treatments, PAW could be operated in batch-type for commercial process. In recent years, it has been widely used for microbial inactivation due to its high bactericidal efficiency, environmental friendliness, ease of preparation, and cost effectiveness (13). Furthermore, PAW exhibits favorable permeability characteristics, enabling it to effectively infiltrate minuscule voids and crevices within food substances, consequently enhancing its disinfectant properties (14). In the study by Gan et al. (15), PAW was employed to treat *Escherichia coli* and *Staphylococcus aureus* on blueberries, demonstrating its effectiveness in reducing microorganisms. In another study by Liu et al. (16), PAW treatment destroyed the cell wall of *Shewanella putrefaciens*, leading to an increase in cell membrane permeability, efflux of intracellular proteins and nucleic acids, and ultimately cell death. These findings highlight the potential effectiveness of PAW for bactericidal applications. However, to our knowledge, the bactericidal mechanism of PAW against *V. parahaemolyticus* has not been fully elucidated. Therefore, this study aimed to investigate the bactericidal effect of PAW against *V. parahaemolyticus*, systematically assess its bactericidal potential and analyze its mechanism by using various parameters, including the cell membrane morphology, cell membrane permeability, intracellular reactive oxygen species (ROS) level, and the leakage of nucleic acids and proteins.

## 2 Materials and methods

### 2.1 Bacteria preparation

*Vibrio parahaemolyticus* (CICC 25008) was purchased from China Industrial Microbial Strain Preservation and Management Centre. *V. parahaemolyticus*, initially stored at −80°C, was revived by inoculation onto thiosulfate citrate bile salts sucrose agar culture medium (TCBS) using the plate streaking method. Subsequently, after overnight incubation at 30°C, a single pure colony was selected for further culturing in 9 mL of alkaline peptone water containing 3% NaCl (w/v). The broth was cultured in a shaking incubator at 30°C for 8 h with a speed of 180 r/min. Activation was performed consecutively twice for a period of 16 h. Then, the broth was centrifuged at 4°C for 10 min with a speed of 8,000 r/min. The supernatant was discarded, and the pellet was resuspended with 0.85% saline to adjust the OD_600_ to a range between 0.8 and 1.0, which refers to the concentration of 10^8^–10^9^ CFU/mL. Triplicate samples were prepared and set aside for subsequent experiments.

### 2.2 Plasma device and plasma-activated water preparation

The PAW was prepared using the CTP-K plasma generator (Suman Electronics Co., Ltd., Nanjing, China) powered for dielectric barrier discharge (DBD) under the following operating conditions, air was utilized as the working gas, with a frequency of 9 kHz, and a power of 200 W. The discharge time ranged from 5, 10, 20, and 30 min was used to produce PAW, denoted as PAW 5, PAW 10, PAW 20, and PAW 30. To maintain the temperature, the PAW was subsequently immersed in an ice bath to reduce the temperature to 4 ± 1°C. Subsequently, the resulting PAW was promptly used for disinfection experimentation.

### 2.3 Analysis of the active compound in PAW

The spectrophotometry was used to measure the concentrations of nitrate anion (NO_3_^−^) and nitrite anion (NO_2_^−^) in PAW (17). Five mL of PAW was transferred to a centrifuge tube and 1 mL of hydrochloric acid (1 mol/L) and 100 μL of sulfamic acid (0.8%) were added. The concentration of NO_3_^−^ in PAW was determined by UV–visible spectrophotometer (Shimadzu UV 2550, Shimadzu Suzhou Instrument Manufacturing Co., Suzhou, China) at a specific wavelength of 220 nm according to the established standard curve of NO_3_^−^.

Forty mL of PAW was transferred to a centrifuge tube and 2 mL of p-aminobenzenesulfonic acid (4 g/L) and 1 mL of naphthylenediamine hydrochloride (2 g/L) were added. The concentration of NO_2_^−^ in PAW was determined by UV–visible spectrophotometer at a single wavelength of 538 nm according to the established standard curve of NO_2_^−^.

The concentrations of hydrogen peroxide (H_2_O_2_) were analyzed using the test Kit (Suzhou Grace Biotechnology Co., Suzhou, China). The electricity conductivity (EC) was determined using a DDB-303A portable conductivity meter (DDB-303A, Shanghai Yidian Scientific Instruments Co., Shanghai, China), and oxidation–reduction potential (ORP) was determined using a SX-630 pen ORP meter (SX-630, Shanghai San-Xin Instrumentation Factory, Shanghai, China). pH value was determined using a digital pH meter (pH-25, Shanghai Kang Yi Instrument Co., Shanghai, China).

### 2.4 Bactericidal effects of PAW against *Vibrio parahaemolyticus*

**Figure 1:**
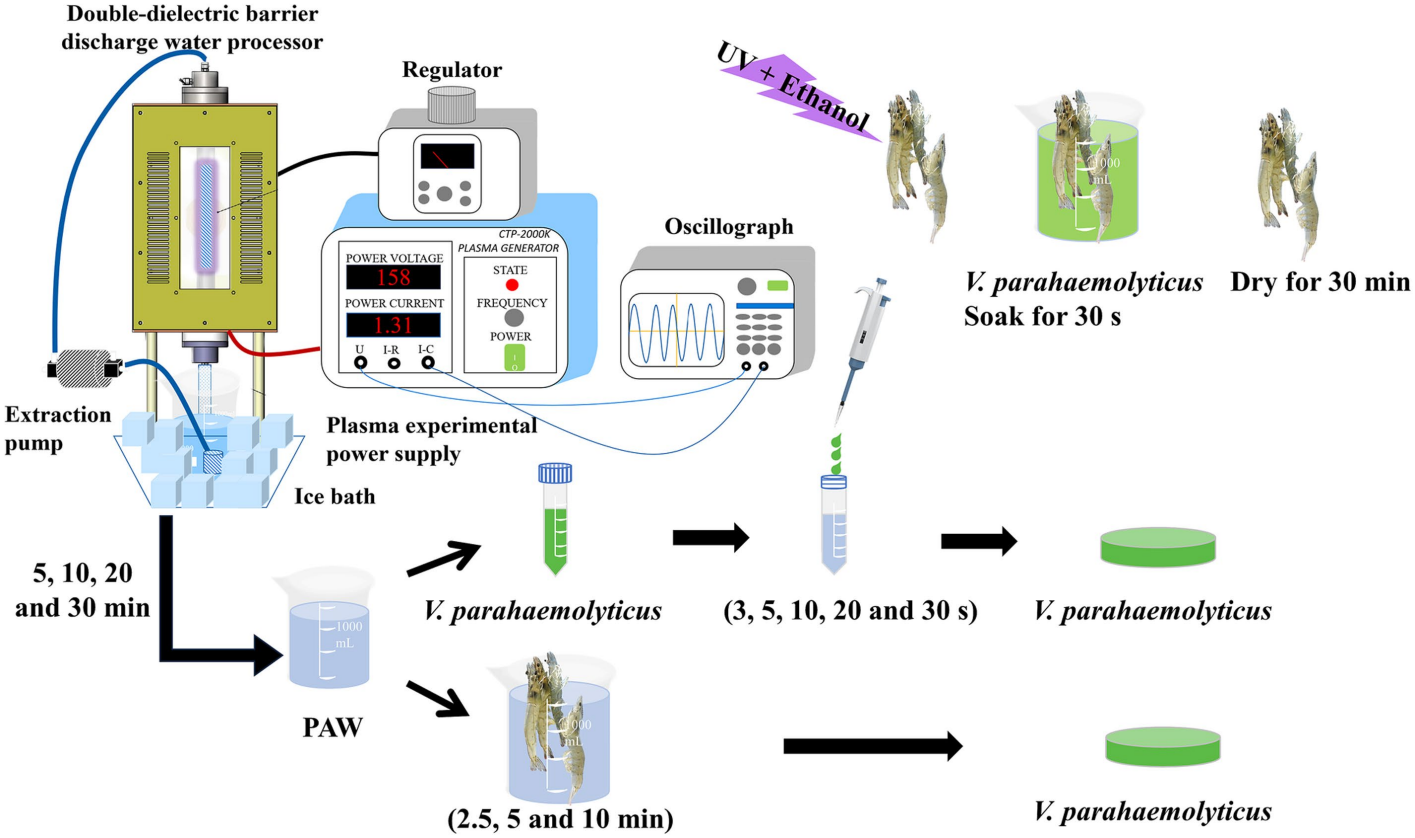
A schematic diagram of plasma double-dielectric barrier discharge and the experimental arrangement, including plasma-activated water (PAW) generation, PAW treatment of *V. parahaemolyticus*, *Litopenaeus vannamei* inoculation with *V. parahaemolyticus* and PAW treatment of inoculated *Litopenaeus vannamei*.

Nine mL of PAW (PAW 5, PAW 10, PAW 20, and PAW 30) prepared at different discharge times were incubated with 1 mL of bacterial suspension at room temperature for different durations (0, 3, 5, 10, 20, and 30 s). The experimental design is shown in Figure 1. Subsequently, gradient dilutions were made, and 100 μL of each bacterial suspension was plated onto chloride tryptic soy agar containing 3% sodium. The plates were incubated for 24 h at 30°C, and the viable colonies were counted. Then, the reductions of *V. parahaemolyticus* in count were calculated.

### 2.5 Cell membrane integrity

#### 2.5.1 Observation of cell morphology

After treating *V. parahaemolyticus* with PAW 20 and PAW 30 for varying durations (0, 3, 10, and 30 s), bacterial cells were collected by centrifugation (3-30 KS, Sigma, Germany) at 4°C for 10 min at 8000 r/min. The cells were then fixed overnight at 4°C in 1 mL of 2.5% (v/v) glutaraldehyde. After washing twice with PBS, a series of ethanol concentrations (30, 50, 70, 90, and 100%) were employed to dehydrate the cells. Subsequently, they were freeze-dried overnight using a freeze-dryer (FDU-1100, Shanghai, China). After being glued onto a sticky stage and gold sprayed, the bacterial powder was observed under a scanning electron microscope (S3400, Hitachi, Tokyo, Japan) at a magnification of 20,000×.

#### 2.5.2 Detection of the leakage of nucleic acids and proteins

The nucleic acid and protein leakage rates of bacterial solutions were determined after PAW treatments (0, 3, 5, 10, 20, and 30 s) (18). The samples were centrifuged at 4°C for 10 min with a speed of 8,000 r/min, and pure water was used as the reference. The leakage of nucleic acids and proteins were measured using a UV-2550 UV–Vis spectrophotometer (Shimadzu, Kyoto, Japan), and the absorbance values were measured at 260 nm and 280 nm, respectively. The following Equations (1) and (2) were used to determine the leakage ratio,


(1) $$\begin{array}{l} \mathrm{Leakage}\;\mathrm{ratio}\;\left(DNA/RNA\right)=\\ \;{OD}_{260}\left(\mathrm{PAW}-\mathrm{treated}\right)/{OD}_{260}\left(\mathrm{control}\right)\; \end{array}$$

(2) $$\begin{array}{l} \mathrm{Leakage}\;\mathrm{ratio}\;\left(\mathrm{Protein}\right)=\\ {OD}_{280}\left(\mathrm{PAW}-\mathrm{treated}\right)/{OD}_{280}\left(\mathrm{control}\right)\; \end{array}$$

### 2.6 Cell membrane permeability

#### 2.6.1 Fluorescence intensity of N-phenyl-1-naphthylamine

The permeability of *V. parahaemolyticus* outer membrane was assessed by investigating the fluorescence intensity of NPN. After PAW treatment for varying durations (0, 3, 5, 10, 20, and 30 s), the bacterial solution was centrifuged (4°C, 2 min with a speed of 10,000 r/min) and resuspended in 1 mL of PBS buffer (0.1 mol/L, pH 7.2). Then, 20 μL of NPN solution was added into 1 mL of the bacterial suspension to make a final concentration of 10 μmol/L. The mixture was incubated at 25°C for 10 min in darkness. To measure the fluorescence intensity, a multifunctional enzyme marker (Molecular Devices, California, United States) with an excitation wavelength of 350 nm and an emission wavelength of 401 nm was applied. The following Equation (3) was used to calculate the relative fluorescence intensity of NPN.


(3) $$NPN\;\mathrm{relative}\;\mathrm{fluorescence}\;\mathrm{intensity}\;\left(\%\right)=\frac{F_{1}}{F_{0}}\times 100$$

where, *F*_0_ represents the fluorescence intensity of the untreated PAW bacterial suspension, and *F*_1_ represents the fluorescence intensity of the PAW-treated bacterial suspension.

#### 2.6.2 Fluorescence intensity of propidium iodide

PI fluorescence intensity was used to evaluate *V. parahaemolyticus* cell membrane permeability. After PAW treatment for varying durations (0, 3, 5, 10, 20, and 30 s), the bacterial solution was centrifuged (4°C, 2 min with a speed of 10,000 r/min) and then resuspended in 1 mL of PBS buffer (0.1 mol/L, pH 7.2). Then, 20 μL of PI solution was added into 1 mL of the bacterial suspension to make a final concentration 3 μmol/L. The mixture was incubated at room temperature for 30 min in darkness. After centrifuging at 4°C for 2 min at 10000 r/min, the mixture was washed twice with PBS, and resuspended. A multifunctional enzyme marker with an excitation wavelength of 482 nm and an emission wavelength of 635 nm was used to measure fluorescence intensity. The results were expressed as the relative fluorescence intensity using the following Equation (4),


(4) $$\mathrm{PI}\;\mathrm{relative}\;\mathrm{fluorescence}\;\mathrm{intensity}\;\left(\%\right)=\frac{F_{1}}{F_{0}}\times 100$$

where, *F*_0_ represents the fluorescence intensity of the untreated PAW bacterial suspension, and *F*_1_ represents the fluorescence intensity of the PAW-treated bacterial suspension.

#### 2.6.3 Detection of electric conductivity

After PAW treatment for varying durations (0, 3, 5, 10, 20, and 30 s), the bacterial solution was centrifuged at 4°C for 10 min with a speed of 8,000 r/min and then resuspended in 1 mL of PBS buffer (0.1 mol/L, pH 7.2). The EC was measured immediately using an electric conductivity meter (DDB-303A, Shanghai, China).

### 2.7 Oxidative damage to cell membranes

#### 2.7.1 Detection of cellular reactive oxygen species levels

The intracellular levels of ROS were evaluated using 2′,7′-dichlorofluorescent yellow diacetate (DCFH-DA). After PAW treatment for varying durations (0, 3, 5, 10, 20, and 30 s), the bacterial solution was centrifuged at 4°C for 10 min with a speed of 8,000 r/min. Then, the pellet was resuspended using 2 mL of DCFH-DA (10 μmol/L) and the solution was incubated in darkness for 30 min. Following the reaction, the solution was centrifugated at 4°C for 2 min with a speed of 10,000 r/min. The pellet was resuspended using an equal volume of PBS. A fluorescence spectrophotometer was used to detect fluorescence intensity at an excitation wavelength of 488 nm and an emission wavelength of 525 nm.

#### 2.7.2 Detection of superoxide dismutase activity

The enzyme activity of SOD was measured according to the kit instructions (Solarbio, Beijing, China). After treatment, the bacterial solution was centrifuged at 4°C for 10 min with a speed of 8,000 r/min. Then, the pellet was resuspended using 1 mL of extract solution in the kit. The mixture was sonicated and centrifuged at 4°C for 10 min with a speed of 8,000 r/min. The supernatant was used to measure the absorbance at 560 nm.

#### 2.7.3 Detection of expression levels of *emrD*, *luxS*, *ompW*

Quantitative Real-Time Polymerase Chain Reactions (qPCR) were used to determine the expression levels of target genes within related biofilms. To verify changes in the expression of biofilm-related genes in the treatment groups compared with the control group, target genes (*emrD*, *luxS*, *ompW*) were selected (19).

The RNA extraction kit (Sangon Biotech, Shanghai, China) was used to extract total RNA from the sample cells, which was then quantified using an ELISA (Molecular Devices, California, United States). RNA yield and integrity were assessed through absorbance and agarose gel electrophoresis, respectively. To generate complementary DNA (cDNA), cDNA Synthesis Kit Ver 2 (Tsingke, Beijing, China) was used for random reverse transcription. For each qCR reaction, the mixture comprised 25 μL of SYBR Green Dye Method Mix (2×), 2.5 μL of each primer (100 μM), 2.5 μL of cDNA, and 17.5 μL of ddH_2_O. The cycling parameters were set as follows: an initial denaturation step at 95°C for 1 min, followed by 40 cycles of denaturation at 95°C for 15 s, annealing at 60°C for 15 s, and extension at 72°C for 30 s. Subsequently, a melting curve analysis of 95°C for 5 s and 60°C for 1 min was performed, followed by a gradual increase in temperature from 60°C to 95°C at a rate of 0.15°C per second while continuously collecting fluorescence data. The primers were synthesized by Bioengineering Co, China and the sequences were presented in Table 1. The changes in relative gene expression were calculated with the 2^−ΔΔCT^ method using the following Equations (5), (6), and (7):


(5) $$\Delta Ct={Ct}_{\mathrm{target gene}}-{Ct}_{\mathrm{reference gene}}$$

(6) $$\Delta \Delta Ct=\Delta {Ct}_{\mathrm{treated sample}}-\Delta {Ct}_{\mathrm{control sample}}$$

(7) $$\mathrm{Relative expression}=2^{-\Delta \Delta Ct}$$

**Table 1:** Primers used for qPCR to evaluate related gene expression.

Genes	Primer	Sequence (5′-3′)
*16S rRNA*	Forward	TTAAGTAGACCGCCTGGGGA
	Reverse	GCAGCACCTGTCTCAGAGTT
*ompW*	Forward	GCAGTCTAGCAGTGGTTGCT
	Reverse	CATTTGGAACGACAGACGCC
*luxS*	Forward	CACTGCGCCTAACAAAGACATT
	Reverse	AAACCAGTGCGACATCCCAT
*emrD*	Forward	TTGCTCCGTTCCCTTACCAC
	Reverse	CATCAAAACACCAAGCGGCA

### 2.8 Bactericidal efficacy of PAW against *Vibrio parahaemolyticus* on *Litopenaeus vannamei*

*Litopenaeus vannamei* with an average weight of 20 ± 0.5 g were obtained from the Xiashan seafood market (Zhanjiang, China). Fresh shrimps were immersed in 75% alcohol for 30 s, washed in sterile water for 2–3 times, and then exposed to UV light on a sterile bench to ensure complete alcohol evaporation (20). The aseptic shrimps were soaked in *V. parahaemolyticus* bacterial solution at a concentration of 10^8^ CFU/mL for 30 s, drained in an aseptic environment, and then transferred to sterile tinfoil for an additional 30 min to allow bacterial adherence. All procedures were performed at room temperature, and inoculated shrimps were used for subsequent experiments.

The shrimps were immersed in 250 mL of PAW 5, PAW 10, PAW 20, and PAW 30 for 2.5, 5, and 10 min, respectively. Afterward, each set of 15 shrimps were picked out, drained, and divided into 5 groups. Sterile water immersion treatment was used as a control. After removing the head, tail, and thread, 5 g of shrimp meat was homogenized for 1 min. The homogenate was sequentially diluted with saline. Subsequently, 1 mL of the appropriate dilution was pipetted into a flat dish containing 3% sodium chloride TSA. The solidified plates were inverted and incubated for 24 h at 37°C.

### 2.9 Statistical analysis

Each experiment was done in triplicates, and the results are presented as the mean ± standard deviation. The JMP 10.0 software (SAS, North Carolina, United States) was utilized to analyze data for each indicator through one-way analysis of variance (ANOVA). Tukey’s test was used to compare the means. The figures were generated with the Origin 2023 software (Origin Lab, Northampton, Massachusetts, United States). The threshold for statistical significance was set at a *p*-value of <0.05.

## 3 Results and discussion

### 3.1 Characterization of PAW prepared under different discharge time

The efficiency of PAW could be measured by pH, EC, ORP, and concentration of ROS and RNS (21). According to Table 2, as the discharge time increased from 0 to 30 min, pH decreased from 6.32 to 4.72, and EC increased from 10.22 to 21.80 μs/cm, while ORP increased from 140.83 to 373.83 mV. After extending the discharge time from 0 to 30 min, the concentration of NO_3_^−^ significantly increased (*p* < 0.05) from 0.20 to 0.59 mg/L, and the concentration of NO_2_^−^ increased from 0.06 to 49.89 mg/L. Additionally, the concentration of H_2_O_2_ increased from 0.09 to 2.55 mg/L. These results aligned with Wang et al. (22), who reported a significant increase (*p* < 0.05) in the ORP, EC, NO_3_^−^, and NO_2_^−^ content, and a decrease in the pH of the PAW. These active substances play a significant role in disinfection and antimicrobial activity. The combined action of ROS and RNS could induce oxidative stress in microbial cells, leading to the disruption of bacterial cell membrane, which further damage intracellular components, such as DNA, RNA, and proteins. In addition, low pH and high ORP also contribute to bacterial inactivation (23). These results showed that the chemical and physical properties of PAW increased when the discharge time increased from 0 to 30 min.

**Table 2:** Change in physical and chemical properties of plasma-activated water under different discharge time.

Factor	Discharge time (min)				
	0	5	10	20	30
pH	6.32 ± 0.02^a^	5.53 ± 0.11^b^	5.47 ± 0.02^b^	5.15 ± 0.13^c^	4.72 ± 0.01^d^
EC (μs/cm)	10.22 ± 0.01^e^	12.60 ± 0.01^d^	14.69 ± 0.01^c^	16.35 ± 0.00^b^	21.80 ± 0.01^a^
ORP (mV)	140.83 ± 10.19^d^	255.67 ± 6.09^c^	276.67 ± 1.37^b^	276.50 ± 8.55^b^	373.83 ± 10.40^a^
H_2_O_2_ (mg/L)	0.08 ± 0.09^d^	2.41 ± 0.06^ab^	2.28 ± 0.07^bc^	2.17 ± 0.15^c^	2.55 ± 0.12^a^
NO_2_^−^ (mg/L)	0.06 ± 0.04^d^	0.44 ± 0.05^d^	8.39 ± 0.09^c^	19.47 ± 0.16^b^	49.89 ± 0.72^a^
NO_3_^−^ (mg/L)	0.20 ± 0.05^d^	0.31 ± 0.08^c^	0.28 ± 0.02^c^	0.44 ± 0.02^b^	0.59 ± 0.04^a^

The results are expressed as mean ± standard deviation (*n* = 3). Values with different letters are significantly different (*p* < 0.05).

### 3.2 The antibacterial efficacy against *Vibrio parahaemolyticus*

**Figure 2:**
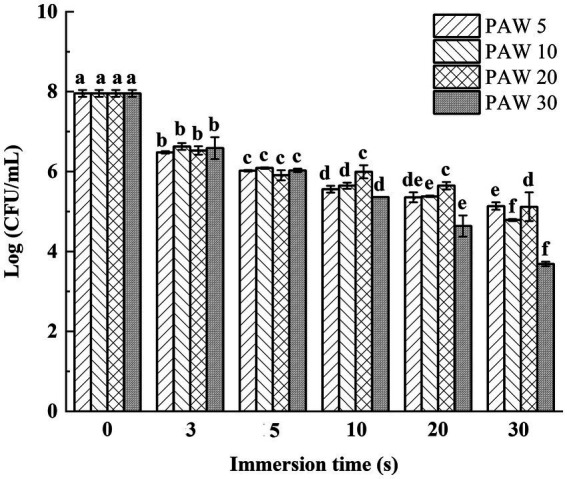
The effects of PAW treatment on viable bacteria count of *V. parahaemolyticus*. PAW 5, plasma-activated water discharge time 5 min; PAW 10, plasma-activated water discharge time 10 min; PAW 20, plasma-activated water discharge time 20 min; PAW 30, plasma-activated water discharge time 30 min. Different lowercase letters above the bars indicate significant differences among groups (*p* < 0.05).

The effects of plasma discharge time and PAW immersion time on the viable bacterial count of *V. parahaemolyticus* are shown in Figure 2. The results demonstrated that the viable bacterial count of *V. parahaemolyticus* dramatically reduced (*p* < 0.05) after immersion in PAW compared to the control group. Notably, after immersion for 30 s, lower viable bacterial counts of *V. parahaemolyticus* were obtained in PAW 5, PAW 10, PAW 20, and PAW 30 than the control group by a reduction of 2.1, 2.7, 3.3, and 4.4 log CFU/mL, respectively. Similar significant decrease (*p* < 0.05) in *Bacillus cereus* endospore after PAW treatment was reported (24). Prolonged plasma discharge time contributed to the increase of ROS and reactive nitrogen species (RNS) in PAW, which are the main factors for bacterial inactivation (25). RNS further decreased the pH of PAW and increased the sensitivity of microorganisms, resulting in microbial inactivation.

### 3.3 Cell membrane integrity

#### 3.3.1 Effect of PAW treatment on cell morphology of *Vibrio parahaemolyticus*

**Figure 3:**
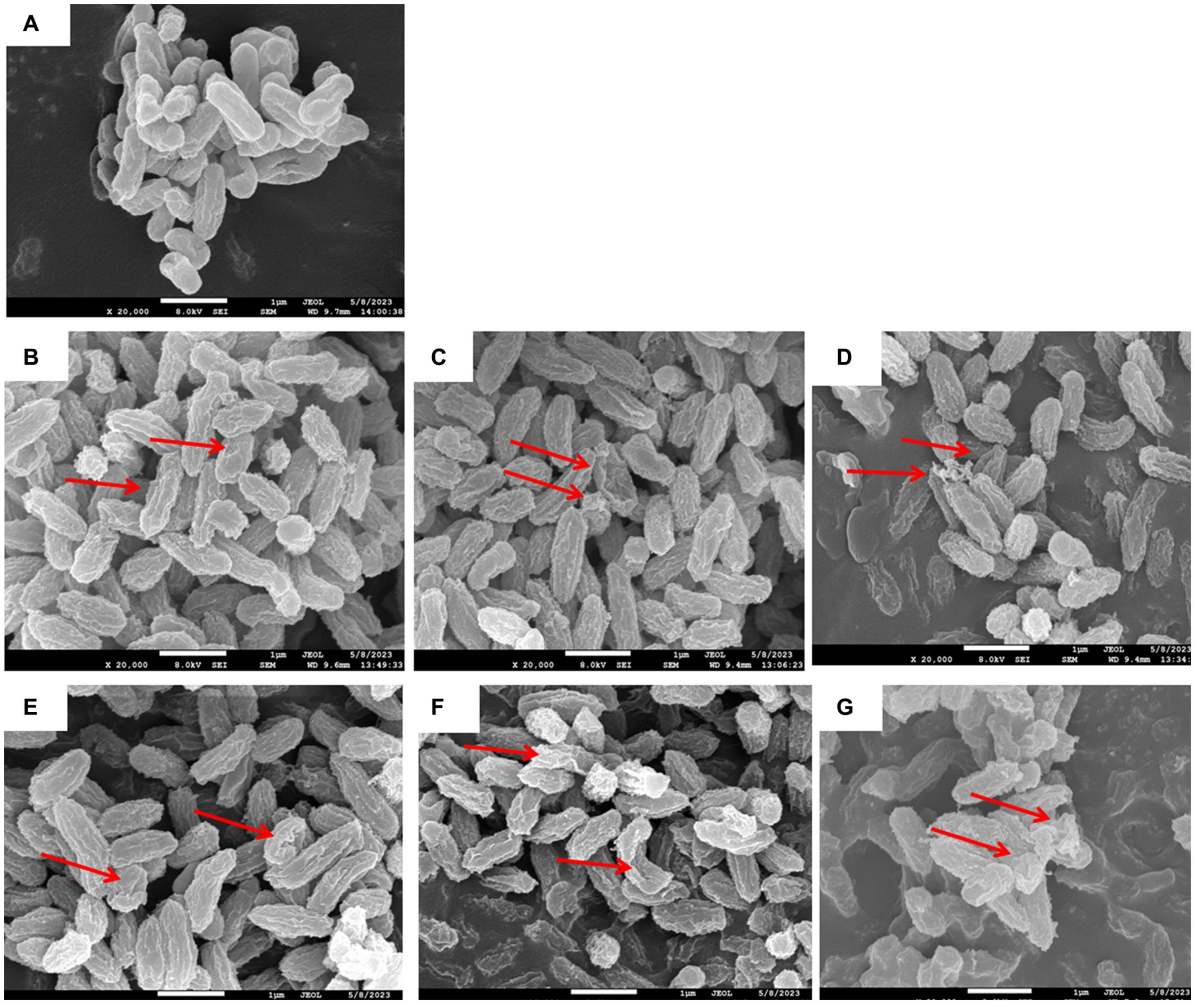
Effects of PAW treatment on cell morphology of *V. parahaemolyticus*. **(A)** Control without any treatment; **(B)** PAW 20 immersion for 3 s; **(C)** PAW 20 immersion for 10 s; **(D)** PAW 20 immersion for 30 s; and **(E)** PAW 30 immersion 3 s; **(F)** PAW 30 immersion 10 s; **(G)** PAW 30 immersion 30 s.

Changes in the cell morphology of *V. parahaemolyticus* after PAW treatment are shown in Figure 3. Scanning electron microscopy (SEM) images revealed that the surface of the untreated group of *V. parahaemolyticus* cells displayed an intact, well-defined, rod-shaped structure with clear boundaries. However, after PAW treatment, *V. parahaemolyticus* cells underwent severe deformation, resulting in a shriveled, irregular appearance. The cell surfaces displayed marked wrinkling, and immersion for 30 s resulted in noteworthy leakage of intracellular solutes. These findings were similar with studies by Liu et al. (26), who reported cell surface damage and disruptions following PAW treatment. The cell surface appeared to be obviously wavy and deep “holes.” These results demonstrate that PAW effectively disrupts cell membrane structures, leading to the leakage of intracellular components and bacterial inactivation.

#### 3.3.2 Effect of PAW treatment on the leakage of nucleic acids and proteins of *Vibrio parahaemolyticus*

**Figure 4:**
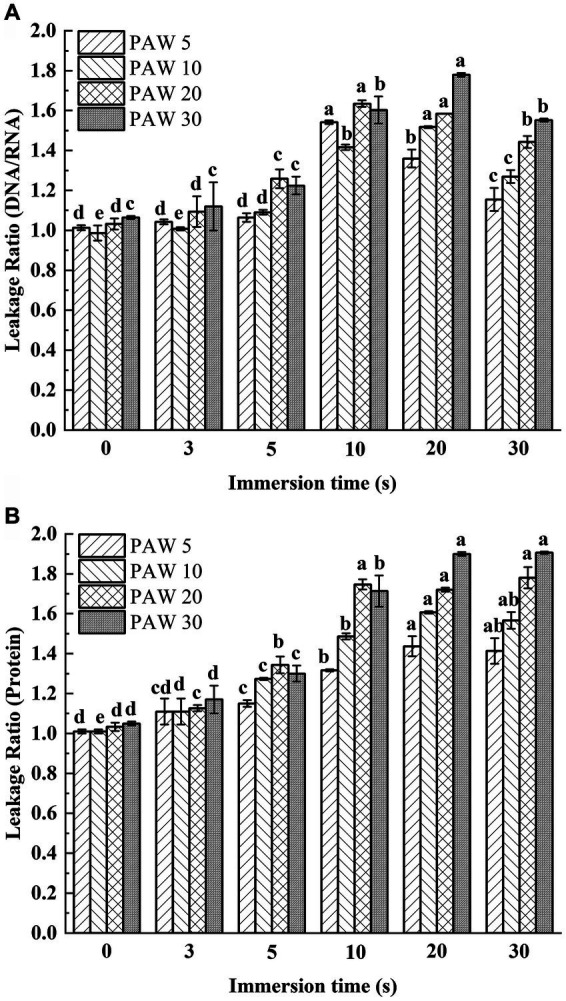
Leakage ratio of nucleic acids and proteins changing with PAW treatment in *V. parahaemolyticus*. **(A)** Optical density (protein). **(B)** Optical density (DNA/RNA ratio). PAW 5, plasma-activated water discharge time 5 min; PAW 10, plasma-activated water discharge time 10 min; PAW 20, plasma-activated water discharge time 20 min; PAW 30, plasma-activated water discharge time 30 min. Different lowercase letters above the bars indicate significant differences among groups (*p* < 0.05).

The leakage of nucleic acids and proteins from cells are general indicators of microbial damage. The extravasation of intracellular substances is a good criterion for studying the integrity of cell membranes. When the bacterial cells are damaged, the internal solution leaked out of the cells. Therefore, the extent of cellular damage can be determined by measuring the leakage rate of nucleic acids and proteins in the supernatant. To assess cell membrane integrity, the leakage of nucleic acids and proteins of *V. parahaemolyticus* exposed to PAW treatment was measured (Figures 4A,B). The leakage rates of proteins and nucleic acids increased with longer plasma discharge time and immersion time compared with the control group (*p* < 0.05). With longer plasma discharge time and immersion time, the leakage ratio of DNA/RNA significantly increased from initial value 1.06 to 1.78 for PAW 30 (*p* < 0.05), and the leakage ratio of proteins significantly increased from initial value 1.05 to 1.91 for PAW 30 (*p* < 0.05). Studies have shown that PAW contains many active substances, such as H_2_O_2_, O_3_, NO_2_^−^, and NO_3_^−^, among others (27). These active substances penetrate bacterial cells, damaging cell membranes, and inducing oxidative damage to nucleic acids, proteins, and other cellular components (28, 29). Xiang et al. (18) also reported a significant increase in intracellular leakage of nucleic acids and proteins with prolonged PAW treatment times. Thus, PAW destroyed the cellular membrane of *V. parahaemolyticus* and caused the leakage of intracellular solutes that becomes more pronounced with longer immersion times.

### 3.4 Cell membrane permeability

#### 3.4.1 Effect of PAW treatment on the outer membrane permeability of *V. parahaemolyticus*

The outer membrane of Gram-negative bacteria is crucial when it comes to adapting to various ambient pressure such as heat, acid, and antibiotics. To assess the impact of PAW on outer membrane permeability, the intracellular fluorescence intensity of NPN was measured (Table 3). NPN is a fluorescent probe that fluoresces strongly in non-polar or hydrophobic environments, making it suitable for evaluating Gram-negative bacterial outer membrane damage (26). The results demonstrated that the fluorescence intensity of NPN increased with longer plasma discharge time and PAW immersion time. After treatment of PAW 30 for 3, 5, 10, 20, and 30 s, the fluorescence intensity of NPN increased by 110.81, 130.04, 135.01, 136.35, and 148.82%, respectively. These findings suggest that PAW treatment destroyed the outer membrane of *V. parahaemolyticus* cells as compared with the control group.

**Table 3:** Change in the electric conductivity, relative fluorescence intensity of NPN, relative fluorescence intensity of PI of PAW under different discharge time.

Factor	Treatment	Immersion time (s)					
		0	3	5	10	20	30
Relative fluorescence intensity of NPN (%)	PAW-5	101.81 ± 2.26^Ac^	106.53 ± 3.19^ABc^	110.44 ± 4.55^Bbc^	118.84 ± 1.77^Bb^	119.71 ± 1.09^Bb^	130.44 ± 5.74^Ba^
	PAW-10	100.53 ± 0.53^Ad^	101.63 ± 1.76^Ad^	121.79 ± 2.47^Ac^	133.61 ± 2.22^Ab^	135.07 ± 0.57^Ab^	142.58 ± 4.66^Aa^
	PAW-20	101.06 ± 0.53^Ae^	110.46 ± 1.37^Ad^	124.89 ± 5.19^Ac^	135.23 ± 0.76^Ab^	136.45 ± 0.85^Aab^	143.26 ± 4.55^Aa^
	PAW-30	102.47 ± 1.33^Ad^	110.81 ± 3.52^Ac^	130.04 ± 4.46^Ab^	135.01 ± 1.55^Ab^	136.35 ± 4.48^Ab^	148.82 ± 2.02^Aa^
Relative fluorescence intensity of PI (%)	PAW-5	101.15 ± 0.77^Ad^	129.62 ± 2.31^Ac^	142.31 ± 3.08^Bb^	145.64 ± 0.44^Bb^	176.03 ± 2.89^Ba^	171.79 ± 5.12^Ba^
	PAW-10	101.15 ± 1.39^Ae^	153.97 ± 3.49^Ad^	159.74 ± 2.99^Acd^	169.36 ± 7.14^Abc^	186.28 ± 2.73^Aa^	179.23 ± 2.77^Aab^
	PAW-20	101.03 ± 0.59^Ad^	150.77 ± 0.67^Ac^	168.33 ± 4.70^Ab^	178.85 ± 1.39^Aa^	183.85 ± 1.54^ABa^	182.56 ± 3.87^ABa^
	PAW-30	101.41 ± 1.35^Ad^	144.21 ± 0.12^Ac^	169.87 ± 6.41^Ac^	172.69 ± 5.55^Abc^	181.67 ± 4.51^ABab^	185.26 ± 2.12^Ab^
EC of the bacterial suspension (μs/cm)	PAW-5	1232.67 ± 6.66^Ae^	1440.33 ± 16.77^Bd^	1516.00 ± 5.00^Bc^	1577.67 ± 7.51^BCb^	1603.67 ± 11.59^BCb^	1638.33 ± 6.81^Ba^
	PAW-10	1230.33 ± 5.51^Ae^	1435.67 ± 12.66^Bd^	1510.00 ± 3.61^Bc^	1571.67 ± 5.03^Cb^	1584.67 ± 3.51^Cb^	1635.33 ± 9.71^Ba^
	PAW-20	1231.33 ± 5.69^Ae^	1452.00 ± 14.53^ABd^	1513.00 ± 13.11^Bc^	1595.33 ± 10.50^Bb^	1605.67 ± 6.66^Bb^	1720.33 ± 15.7^Aa^
	PAW-30	1230.33 ± 5.51^Ae^	1486.67 ± 10.02^Ad^	1541.33 ± 7.51^Ac^	1635.33 ± 5.13^Ab^	1636.67 ± 8.02^Ab^	1748.67 ± 15.14^Aa^

The results are expressed as mean ± standard deviation (*n* = 3). Values with different letters are significantly different (*p* < 0.05). Different capital letters showed significant differences in different soaking time (*p* < 0.05). Different lowercase letters indicated significant differences in different PAW treatments (*p* < 0.05).

#### 3.4.2 Effect of PAW treatment on the inner membrane permeability of *Vibrio parahaemolyticus*

The permeability of the inner membrane was assessed using PI, which can penetrate damaged cell membranes and bind to DNA, emitting red fluorescence when excited at 540 nm (30). As shown in Table 3, the relative fluorescence intensity of PI in the PAW-treated group was generally higher than that in the control group. With the increase of plasma discharge time, the relative fluorescence intensity of PI in the PAW treatment group increased gradually (*p* < 0.05), indicating a time-dependent increase in cell membrane penetration. This observation is consistent with the study by Liu et al. (16), who indicated that the interaction between ROS and RNS in PAW may destroy the membrane. However, further investigation is needed to identify the specific active substances and interactions for membrane disruption.

#### 3.4.3 Effect of PAW treatment on the electric conductivity of *Vibrio parahaemolyticus*

The cell membrane is an important protective barrier for bacterial cells. When bacterial cells are in an unfavorable environment, the cell membrane becomes damaged, resulting in the change in cell membrane permeability. This is followed by the leakage of internal electrolytes and increased EC (31). Therefore, the change in cell membrane permeability can be reflected by measuring the change in EC of the bacterial suspension. As illustrated in Table 3, EC increased with longer plasma discharge time and immersion time (*p* < 0.05). The highest conductivity was observed after 30 s of PAW immersion. The increase in EC is attributed to the damage to the cell membrane, which caused leakage of intracellular electrolytes into the solution, ultimately increasing conductivity.

### 3.5 Oxidative damage of cell membrane

#### 3.5.1 Effect of PAW treatment on intracellular ROS of *Vibrio parahaemolyticus*

**Figure 5:**
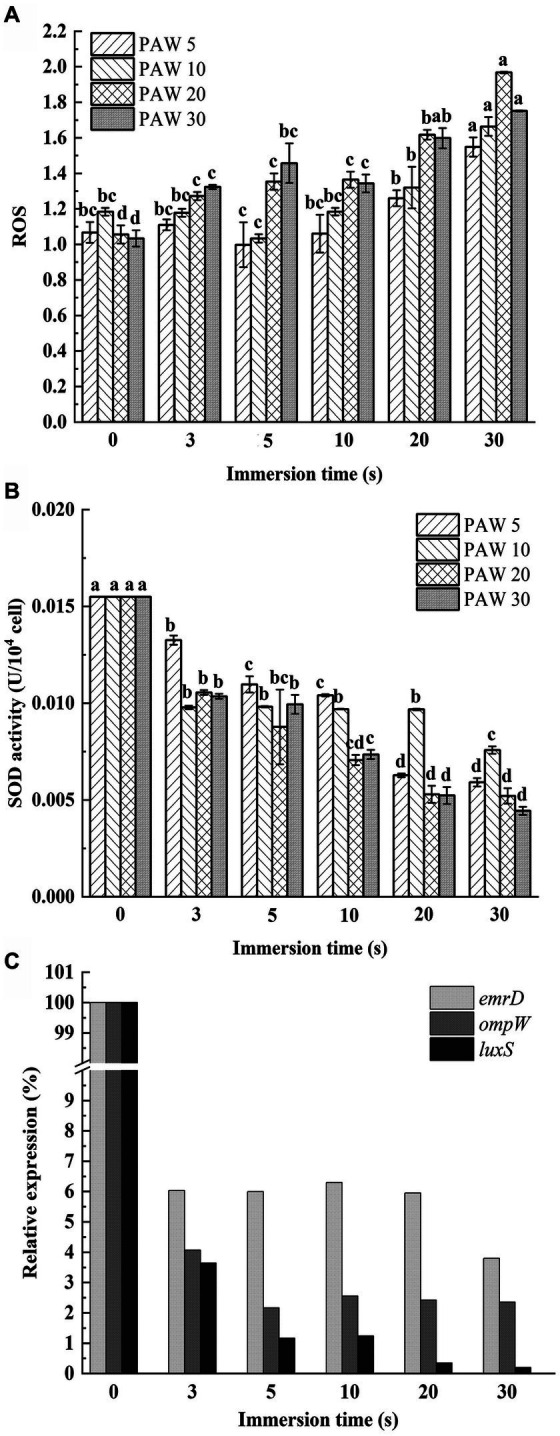
Effects of PAW treatment on oxidative damage of cell membrane of *V. parahaemolyticus*. **(A)** The relative expression level of intracellular reactive oxygen species (ROS). **(B)** SOD enzyme activities. **(C)** The relative expression levels of the *ompW*, *emrD*, and *luxS* genes. PAW 5, plasma-activated water discharge time 5 min; PAW 10, plasma-activated water discharge time 10 min; PAW 20, plasma-activated water discharge time 20 min; PAW 30, plasma-activated water discharge time 30 min. Different lowercase letters above the bars indicate significant differences among groups (*p* < 0.05).

The DCFH-DA probe was utilized to measure intracellular ROS levels in *V. parahaemolyticus* after PAW treatment (Figure 5A). The relative expression level of intracellular ROS increased dramatically with the increasing plasma discharge time and immersion time (*p* < 0.05). The highest ROS accumulation was observed in *V. parahaemolyticus* cells immersed in PAW 20 for 30 s, with a relative expression level reaching 1.97-fold. These results demonstrate that PAW stimulates high ROS generation in *V. parahaemolyticus* cells, resulting in substantial oxidative damage to the cell membrane. The intracellular ROS are crucial for maintaining cell function and signal transduction. However, when intracellular ROS exceed the cellular antioxidative threshold, large amount of ROS cause irreversible oxidative damage to biomacromolecules including DNAs, proteins, lipids, and enzymatic systems from inside, thereby disrupting the physiological functions of bacterial (32). Additionally, the active substances in PAW, such as H_2_O_2_, O_3_, ONOO^−^, NO_2_^−^, and NO_3_^−^, may induce oxidative injury to bacterial cell membrane lipids and intracellular nucleic acids and proteins, leading to oxidative stress and cell dysfunction (33).

#### 3.5.2 Effect of PAW treatment on SOD activity of *Vibrio parahaemolyticus*

SOD is a metalloenzyme widely found in living organisms and is an important oxygen radical scavenger that catalyzes the disproportionation of superoxide anions to produce H_2_O_2_ and O_2_, which has an important role in the antioxidant system. A gradual decrease in SOD activity was observed with longer plasma discharge time and PAW immersion time (Figure 5B). After 30 s of PAW immersion, SOD activity in *V. parahaemolyticus* treated with PAW 5, PAW 10, PAW 20, and PAW 30 decreased by 61.47, 60.44, 66.50, and 72.74%, respectively. These results indicate that PAW may destroy the antioxidant defense system of *V. parahaemolyticus*, promoting peroxidation process (16).

#### 3.5.3 Effect of PAW treatment on gene expression of *Vibrio parahaemolyticus* cell membranes

To understand the mechanisms of cell membrane damage, the relative expression levels of the *ompW*, *emrD*, and *luxS* genes were evaluated by qRT-PCR (Figure 5C) (6). As an outer membrane protein synthesis gene, *ompW* has functions in disrupting the regular transport of biofilm formation substances, interfering with the early adhesion of bacterial cells, and inhibiting the growth of biofilms (34). As a drug resistance and metabolism-related gene, *emrD* can interfere with metabolic function and reduce drug resistance (35). The population-sensing gene *luxS* is a key gene that affects the normal function of other population-sensing signaling systems (36). As shown in Figure 5C, the relative expression levels of *ompW*, *emrD*, and *luxS* genes of *V. parahaemolyticus* dramatically decreased after PAW treatment (*p* < 0.05). The result suggests that PAW destroy the normal physiological balance in *V. parahaemolyticus* cells by regulating gene expression. This disruption leads to cell membrane damage, impairing normal bacterial function and contributing to enhanced bactericidal efficiency (37).

### 3.6 The bactericidal activity of PAW against *Vibrio parahaemolyticus* on *Litopenaeus vannamei* surface

**Figure 6:**
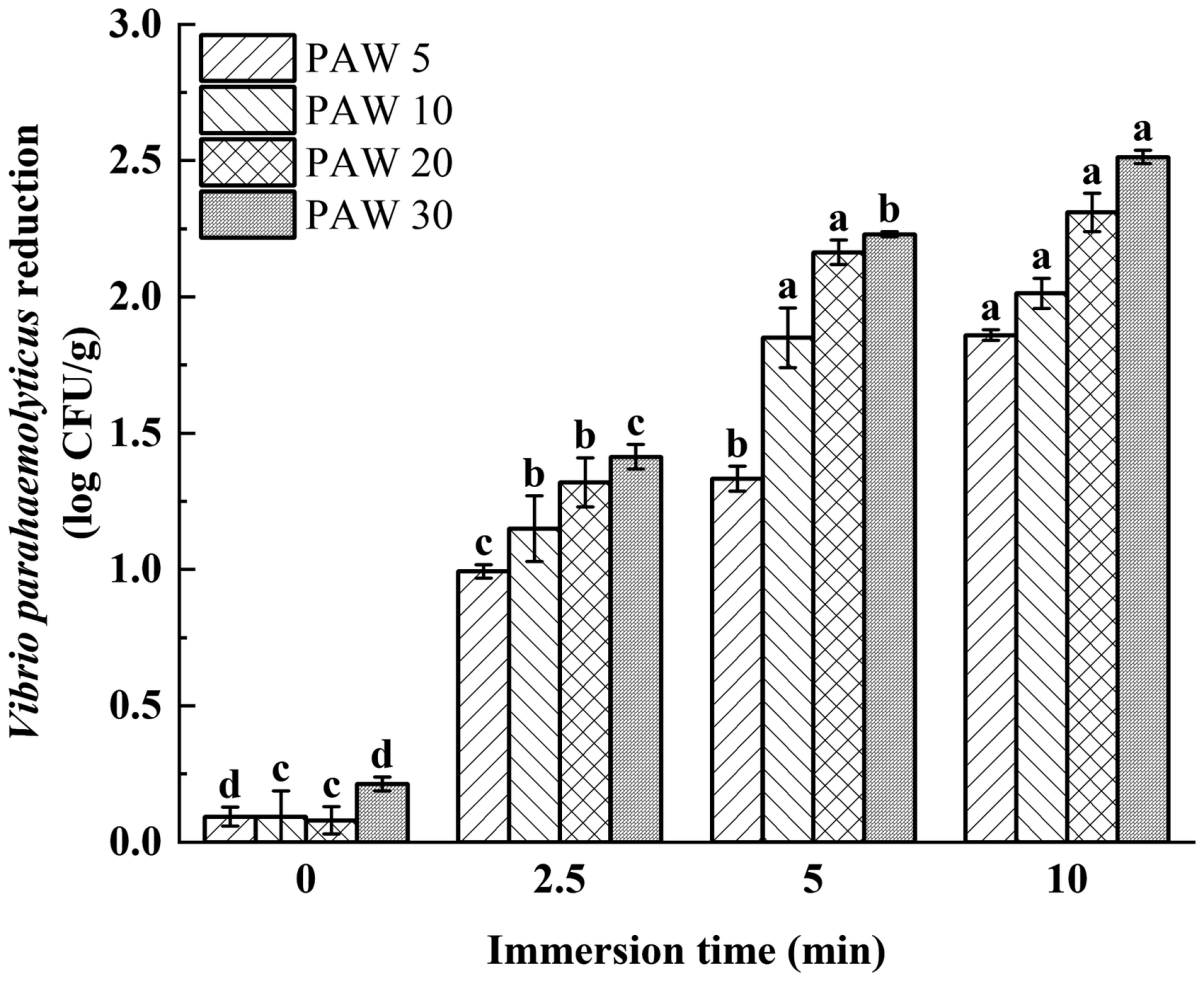
The effects of PAW treatment on viable bacteria count of *V. parahaemolyticus* inoculated on shrimp surface. PAW 5, plasma-activated water discharge time 5 min; PAW 10, plasma-activated water discharge time 10 min; PAW 20, plasma-activated water discharge time 20 min; PAW 30, plasma-activated water discharge time 30 min. Different lowercase letters above the bars indicate significant differences among groups (*p* < 0.05).

*Vibrio parahaemolyticus* was inoculated onto the shrimp surface for evaluation of the bactericidal effect of PAW on *L. vannamei*. The effect of plasma discharge time and PAW immersion time on *V. parahaemolyticus* on the shrimp surface was investigated (Figure 6). The results showed that with the increase of plasma discharge time and prolonged PAW immersion time, the viable *V. parahaemolyticus* count on the surface of *L. vannamei* decreased significantly (*p* < 0.05). After treatment for 5 min, the viable count of *V. parahaemolyticus* on the surface of shrimp decreased by 1.3, 1.8, 2.1, and 2.2 log CFU/g for PAW 5, PAW 10, PAW 20, and PAW 30, respectively. Similar findings were reported (38, 39), indicating with longer plasma discharge time and immersion time, stronger antimicrobial properties. However, it’s important to note that while PAW treatment reduced microbial counts, complete elimination of microorganisms was not achieved, possibly due to the irregular surface of shrimp, which provides refuge for pathogens (40). Thus, to maximize PAW’s effectiveness in the food processing industry, a preliminary water treatment step to remove surface contaminants may enhance the sterilization efficiency of PAW (39).

## 4 Conclusion

In summary, this study has demonstrated the effectiveness of PAW treatment in reducing the viability of *V. parahaemolyticus*. The results indicated a correlation between the physical and chemical characteristics of PAW and its inactivation efficacy. The activity of PAW increased with the increase of discharge time, and the bactericidal efficacy of PAW increased with longer plasma discharge time and immersion time. Notably, PAW 30 treatment group exhibited the highest sterilization efficacy, achieving a 99.9% bacterial inhibition rate after 10 s of treatment. PAW treatment inflicted significant damage to *V. parahaemolyticus* cell membranes, leading to cell deformation, leakage of nucleic acids and proteins, increased membrane permeability, and oxidation stress. PAW treatment made it difficult for *V. parahaemolyticus* to form biofilm, and greatly increased the elimination rate of *V. parahaemolyticus* biofilm. Furthermore, the study explored the application of PAW for reducing *V. parahaemolyticus* on the surface of shrimp, offering potential benefits for food safety and processing.

## Data availability statement

The original contributions presented in the study are included in the article/supplementary materials, further inquiries can be directed to the corresponding author.

## Author contributions

HZ: Data curation, Formal analysis, Methodology, Writing – original draft. JW: Formal analysis, Methodology, Writing – original draft. HX: Formal analysis, Methodology, Writing – original draft. IK: Formal analysis, Writing – review & editing. QS: Data curation, Software, Writing – review & editing. XZ: Data curation, Formal analysis, Writing – review & editing. JG: Data curation, Formal analysis, Writing – original draft. SL: Conceptualization, Funding acquisition, Investigation, Methodology, Project administration, Writing – review & editing. SW: Conceptualization, Funding acquisition, Investigation, Project administration, Writing – review & editing.
